# Disrupted classes, undisrupted learning during COVID-19 outbreak in China: application of open educational practices and resources

**DOI:** 10.1186/s40561-020-00125-8

**Published:** 2020-07-06

**Authors:** Ronghuai Huang, Ahmed Tlili, Ting-Wen Chang, Xiangling Zhang, Fabio Nascimbeni, Daniel Burgos

**Affiliations:** 1 Smart Learning Institute of Beijing Normal University, Beijing, China; 2 UNIR iTED, Universidad Internacional de La Rioja (UNIR), Logroño, Spain

**Keywords:** Coronavirus (COVID-19), Online education, Distance education, Open education, Open educational resources (OER), Open educational practices (OEP)

## Abstract

With the coronavirus (COVID-19) outbreak in China, the Chinese government decided to ban any type of face-to-face teaching, disrupting classes and resulting in over 270 million students being unable to return to their universities/schools. Therefore, the Ministry of Education (MoE) launched an initiative titled ‘Ensuring learning undisrupted when classes are disrupted’ by reforming the entire educational system and including an online education component. However, this quick reform in this unexpected critical situation of widespread COVID-19 cases harbours several challenges, such as the lack of time and teacher/student isolation. This paper discusses the possibility of using open educational resources (OER) and open educational practices (OEP) as an effective educational solution to overcome these challenges. Particularly, this study presents a generic OEP framework built on existing open-practice definitions. It then presents, based on this framework and based on the challenges reported by several Chinese education specialists during two national online seminars, a set of guidelines for the effective use of OER and OEP for both teaching and learning. Finally, this study presents some recommendations for the better adoption of OER and OEP in the future. The findings of this study can help researchers and educators apply OER and OEP for better learning experiences and outcomes during the COVID-19 outbreak.

## Introduction

A pneumonia outbreak was first reported in the city of Wuhan, the capital of Central China’s Hubei province, in December 2019. Experts have attributed the outbreak to a novel coronavirus (COVID-19) whose first four cases are linked to the Huanan (Southern China) Seafood Wholesale Market (Li et al. 2020). Since then, the COVID-19 has spread across China and worldwide, causing over 70,000 deaths. The World Health Organization (WHO 2020) defined *coronavirus* as ‘a large family of viruses that cause illness, ranging from the common cold to more severe diseases, such as Middle East [respiratory syndrome] (MERS-CoV) and [severe acute respiratory syndrome] (SARS-CoV). A novel coronavirus (nCoV) is a new strain that has not been previously identified in humans’.

To contain the COVID-19 spread, the Chinese government issued a notice for all people, including students, to remain home for quarantine until further notice, resulting in almost 276 million students being unable to return to their schools and universities (Huang et al., 2020). UNESCO (2020) highlighted that over 1.5 billion learners around the world were not able to attend school or university due to the COVID-19 outbreak as of April 4th, 2020. In this context, the Chinese Ministry of Education (MoE) and several education specialists and universities have started discussing the use of information and communication technology (ICT) to reform the entire educational system in the midst of this pandemic and provide online and distance learning instead, even with disrupted classes. Generalised online and distance education in China started in the 1960s, when Chinese radio and TVs started offering distance education to remote areas, while several Chinese universities have adopted online education since 1998 (Ting et al. 2018). While online and distance education is not new in China, several challenges have arisen regarding this type of system in this unexpected and critical situation, namely, the following:

1. *Lack of preparation time:* Teachers have not prepared their learning content to adapt to online learning, and preparing such content will take time. Similarly, several universities and schools have not improved their online learning environments to support this kind of learning experience.
2. *Teacher/student isolation:* In this first-ever application of pure long-term online learning (without face-to-face learning or blended learning), both teachers and students should not feel that they are left alone during the teaching and learning processes.
3. *Need for effective pedagogical approaches:* New effective pedagogical approaches are needed to keep students motivated and engaged during this long period of online learning, especially that drop-out rates of distance learning is generally higher than in-campus based learning.

To help overcome the problem of limited time to prepare online learning content, teachers should make use of the thousands of open educational resources (OER) published by the MoE and available in other national and international repositories as well as public online tools, platforms, and enabling technologies. The term *open educational resources* was first coined at UNESCO’s 2002 Forum on Open Courseware, and it was defined in the recent UNESCO recommendation on OER as ‘learning, teaching, and research materials in any format and medium that reside in the public domain or are under copyright that have been released under an open licence that permit no-cost access, [reuse], [repurpose], adaptation, and redistribution by others’ (UNESCO 2019). Blackall and Hegarty (2011) also mentioned that using OER can save time in preparing learning materials.

To solve the problems related to teacher/student isolation as well as the need for effective pedagogical approaches to keep students active and engaged, teachers should build their courses around OER and ask their students to find content to solve problems, write reports, or research on topics. Specifically, open educational practices (OEP) – including open pedagogy, open collaboration, and open assessment – should be implemented to keep the students motivated and engaged during this long period of online learning. Ehlers (2011) defined OEP as ‘practices which support the (re) use and production of OER through institutional policies, promote innovative pedagogical models, and respect and empower learners as co-producers on their lifelong learning paths’. The recently approved UNESCO (2019) OER recommendation also stated that ‘the judicious application of OER, in combination with appropriate pedagogical methodologies, well-designed learning objects, and the diversity of learning activities, can provide a broader range of innovative pedagogical options to engage both educators and learners to become more active participants in educational processes and creators of content as members of diverse and inclusive [knowledge societies]’. Chiappe and Adame (2018) stated that OEP have become a growing educational trend based on ICT.

This study discusses how to apply OER and OEP in the Chinese educational system during this COVID-19 outbreak. Particularly, based on a review of the literature and with the help of several government departments and education specialists, this study offers several guidelines for Chinese educators regarding OER and OEP application in education. Additionally, this study discusses strategies urgently applied by the Chinese government as well as Chinese companies and universities to support open and online learning. Finally, based on the challenges identified by Chinese education specialists and the authors of this study (themselves educators and researchers), several recommendations are presented to enhance the future adoption and application of OER and OEP in China and in other contexts. Specifically, the suggested enabling tools and technologies were identified for both the Chinese and international contexts. Particularly, examples of alternative international enabling tools and technologies were mentioned between “parentheses” for international readers to apply the suggested OEP in their respective contexts. For instance, instead of using Sina Weibo (Chinese social network), these readers can use Twitter or Facebook.

The rest of the paper is structured as follows. Section 2 presents a literature review about OER and OEP in China as well as a generic OEP framework for open education based on several definitions of OEP in the literature. Section 3 presents guidelines on using OER and OEP during this COVID-19 outbreak. Section 4 presents a set of urgent applied strategies to support open and online learning by the Chinese government as well as Chinese universities/schools and companies. Finally, Section 5 offers several recommendations to enhance OER and OEP adoption and application in China, concludes the paper, and presents future directions based on this research.

## Literature review

This section starts by conducting a review on OER and OEP in China. It then presents an OEP framework for open education.

### OER in China

The concept of OER was introduced in China following the MIT OpenCourseWare conference in Beijing in 2003. Since then, the Chinese government has launched several initiatives to enhance OER adoption. For instance, the MoE provided 5 years’ worth of funds to support the Chinese Quality Course (CQC) project, which aims to provide open learning materials to the public for free. Particularly, Chinese universities provide bonus funding for teachers who contribute to the CQC project by publishing their courses within this project. The Chinese Ministry of Culture and Finance also funded the National Cultural Information Resources Sharing Project (NCIRSP), which focuses on sharing cultural resources that aid in the construction of the public service system of culture in China. Finally, the Chinese Ministry of Science and Technology funded the Science Data Sharing Project (SDSP), which focuses on providing open data to the public as well as guidelines and standards on open data, including data collection and publication.

Several institutional initiatives were also launched to support OER adoption. For instance, in 2012, the Open University of China launched the five-minute course initiative, which aims to build 30,000 five-minute courses involving 100 subjects in several fields. Another initiative is XuetangX, launched by Tsinghua University and MOOC-CN Information Technology in 2013, which provides access to over 1000 free courses from Tsinghua, Fudan, MITx, HarvardX, and other universities. Today, open teaching resources in China can be grouped into three categories, where some of them are not in compliance with all OER conditions as specified in the UNESCO OER definition (Tlili, Huang, Chang, Nascimbeni, & Burgos, 2019), namely: resources which are made publicly available by Chinese universities and libraries for free but without any open licences; resources which are under open licences or protected by Chinese copyright laws that allow their free use and/or reuse; and resources which are not under open licences and do not reside in the public domain yet are made available for free public use by government policies.

With the rapid evolution of the open education concept, researchers have shifted their focus from content-centred approaches, which focus on educational resources (creation, sharing, etc.), to more practice-centred ones that foster collaboration between learners and teachers for creating and sharing knowledge (Cronin, 2017). In other words, researchers and educators have shifted their focus from creating and publishing OER to practices that can be implemented using OER for education, referred to as Open Educational Practices (OEP). In line with these developments, this study focuses on OEP that could be used to provide active and engaging learning experiences for learners during this COVID-19 outbreak. To do so, OEP must be fully understood since, as noted by Cronin (2017), their scope is rapidly evolving, and researchers tend to focus on different OEP perspectives. Therefore, the next section aims to draw an OEP framework based on several reported OEP definitions in the literature. The authors will then refer to this framework when presenting guidelines about OEP application in education during the COVID-19 outbreak.

### OEP framework for open education

To better understand OEP, a comprehensive review was conducted about the reported OEP definitions in the literature, as shown in Table 1. Several keywords were then identified from each definition (see Table 1). Finally, based on these keywords, five conditions are identified and discussed below, which have been used to create the OEP framework of this study.

- *OER:* Teaching materials used within OEP should be openly licenced, and the resources produced during the course (e.g. reports, presentations, videos) should also be released as OER.
- *Enabling technology:* Teachers should make use of different technologies and tools to build and support a connected learning community where the OEP can flourish. These technologies and tools include OER authoring tools, OER repositories, social networks, and collaborative editing tools.
- *Open teaching:* Educators should implement teaching methodologies that can help students to construct their own learning pathways (self-regulated) and to actively contribute to knowledge building, both individually and collaboratively.
- *Open collaboration:* Teachers should build open communities, for instance by using social networks, to help students to work in teams to carry out particular learning tasks (e.g. editing a blog, creating a Wikipedia page) as well as to exchange ideas and discussions related to those specific learning tasks. Other teachers and stakeholders can participate in these discussions as well to further assist learners.
- *Open assessment:* Teachers should allow learners to evaluate one another (peer assessment). This can emphasise reflective practices and improve learning outcomes.

**Table 1:** Review of OEP definitions in the literature and the extracted keywords

Definitions	Keywords
The Open eLearning Content Observatory Services (OLCOS) project defined OEP as ‘practices that involve students in active, constructive engagement with content, tools, and services in the learning process and promote learners’ self-management, creativity, and working in teams’ (Geser, 2007).	tools and services, working in teams
Conole and Ehlers (2010) defined OEP as ‘the use of OER with the aim to improve [the] quality of educational processes and innovate educational environments’ (p. 3).	OER, educational environment
Ehlers (2011) considered OEP as a ‘collaborative practice in which resources are shared by making them openly available and pedagogical practices are employed which rely on social interaction, knowledge creation, [peer learning], and shared learning practices’ (p. 6).	collaborative practice, social interaction, knowledge creation, peer learning
International Council for Open and Distance Education (ICDE, 2012) defined OEP as “practices which support the production, use and reuse of high quality open educational resources (OER)”	Practices, (re) use of OER
UK OER support and evaluation team (2012) defined OEP as “all activities that open up access to educational opportunity, in a context where freely available online content and services (whether ‘open’, ‘educational’ or not) are taken as the norm”	open services, open content
Other researchers (DeRosa & Robison, 2015; Hegarty 2015; Rosen & Smale, 2015; Weller, 2014) considered OEP as an open pedagogy where students contribute to the teaching process using OER.	open pedagogy
Cronin (2017) defined OEP as ‘collaborative practices that include the creation, use, and reuse of OER as well as pedagogical practices employing participatory technologies and social networks for [the] interaction, [peer learning], knowledge creation, and empowerment of learners’.	collaborative practices, OER, participatory technologies, social networks, peer learning, knowledge creation
The Ljubljana Action Plan from the Second World OER Congress (UNESCO, 2017) stated, ‘If used effectively and supported by sound pedagogical practices, OER allow for the possibility to dramatically increase access to education through ICT, opening up opportunities to create and share a wider array of educational resources to accommodate a greater diversity of educator and learner needs. Increased online access to OER further promotes individualised study, which, when coupled with social networking and collaborative learning, fosters opportunities for pedagogical innovation and knowledge creation.’	ICT, social networking, collaborative learning, knowledge creation
Institute for the Study of Knowledge Management in Education (2017) defined OEP as “comprising a set of skills in collaboration, curation, curricular design, and leadership around the use of Open Educational Resources. OEP build educator capacity for using OER to improve curriculum, instruction, and pedagogy, and to gain skills in digital resource curation and curriculum creation, and to actively collaborate around and advocate for innovative approaches to open education and OER”	OER use (skills), collaboration, resource and curriculum creation
Chiappe and Adame (2018) stated that OEP can cover several dimensions, including assessment, teaching, and educational planning.	assessment, teaching, educational planning
Wiley and Hilton III (2018) considered OEP as an OER-enabled pedagogy and defined it as a ‘set of teaching and learning practices that are only possible or practical in the context of the 5R permissions that are characteristic of OER’.	teaching and learning practices, OER

**Fig. 1:**
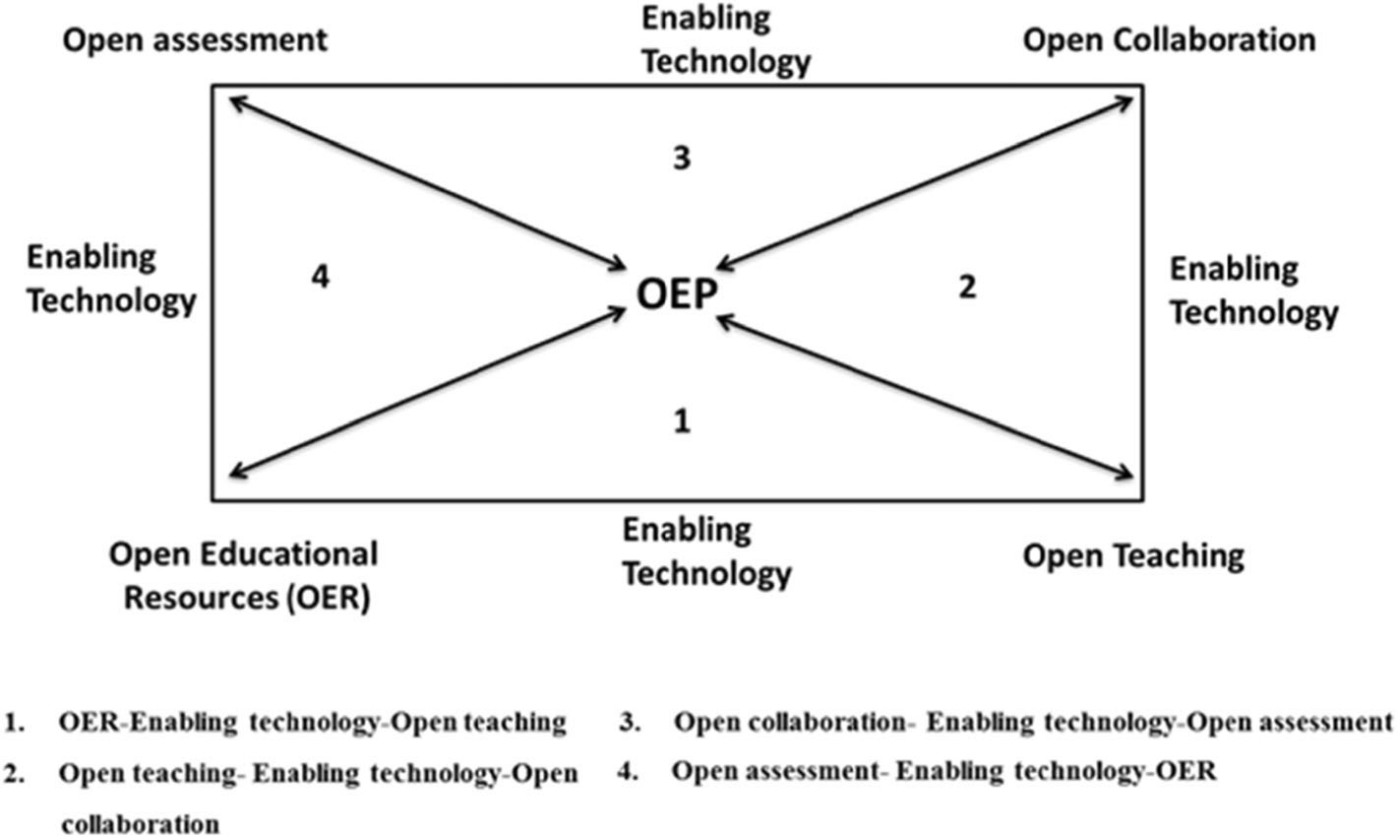
OEP framework for open education

Since OEP, as with all teaching activities, are complex and multipronged practices, the conditions above are interrelated. Specifically, four relations, discussed below, can be formed among the five aforementioned conditions. All these relations are mediated by technology that is conceived as an enabling condition for OEP to be developed, not as the central aspect of the practice. It should be noted that numbers are used as indices of relations and do not reflect the order of importance of these relations. It should also be noted that the presented five conditions and four relations in Fig. 1 can lead to new conditions and relations, however they were not considered as they are not directly related to this study focus, that is to help providing effective educational experience using OER and OEP during COVID-19 outbreak. For instance, all these relations result in a large amount of learning interaction data, that can be released as “open data” to further enrich the educational offer (Atenas et al. 2015).

1. *Relation OER–enabling technology–open teaching:* This is possibly the most typical example of OEP. Thanks to this relation, students can enjoy open and collaborative learning processes based on OER via participatory technology. For instance, teachers can use OER, such as open textbooks, as teaching content and ask their students to further improve such content by remixing it with other content or by creating new activities and exercises for the course.
2. *Relation open teaching–enabling technology–open collaboration:* Through this relation, teachers foster students’ engagement in open collaborations via technology, such as social media and networks, where they engage with existing online and offline communities and stakeholders.
3. *Relation open collaboration–enabling technology–open assessment:* This relation describes the case when students are assessed by relying on collaboration with existing external communities and stakeholders, also resulting in open assessment reports (e.g. dashboards) for everyone.
4. *Relation open assessment–enabling technology–OER:* This regards fostering collaborative and peer assessment of the resources developed individually or in groups by the learners; the obtained open assessment (feedback, reports, and dashboards) can then be used to enhance the OER.

## Challenges and guidelines on using OER and OEP during this COVID-19 outbreak

Two national online seminars on the ‘[first-line] demand and implementation of online and open education during the COVID-19 outbreak’ were organised by the National Engineering Laboratory for Cyberlearning and Intelligent Technology during February 19 and 27, 2020. More than 140 individuals attended these two seminars, 40 of whom were experts and education specialists from several universities and schools (primary, middle, and high school) from different areas and provinces, including remote areas, and Wuhan, the city with the most COVID-19 cases. The goal of these seminars was to gather these experts with various educational experiences in different contexts (urban vs. remote, universities vs. schools, and totally closed areas like Wuhan vs. less closed areas) to discuss the new educational system reform towards open and distance education and the potential challenges that teachers or learners might face. Particularly, five researchers from the Smart Learning Institute of Beijing Normal University (SLIBNU) summarised the challenges reported during this seminar and then grouped them via a card sorting method. This method is used to organise and improve the architecture of the information and has been used in various fields, such as educational robots (Cheng, Sun, & Chen, 2018), to create different groups of collected information. The grouping process was applied by the five researchers based on the definition of each challenge. Particularly, in cases when the grouping was different, an agreement was reached through discussions. Based on the obtained challenges (specifically those related to our study) as well as on other challenges further reported during this COVID-19 outbreak by Chinese scholars and international experts in general (in the literature), several guidelines on using OER and OEP for learning and teaching are discussed below.

### Guidelines for teachers

To ensure an active and engaging teaching experience using OER and OEP for better learning outcomes, teachers should refer to the following guidelines:

- Yang (2020) mentioned that copyright is one of the challenges of using online resources. Indeed, during OEP development, teachers should pay attention to the attributed open licence of each OER to ensure its legal use in their context.
- Professor ID-012 from the online seminar mentioned that teachers might not be familiar with the process of choosing the most suitable resources to use in their teaching processes. In this context, Ozdemir and Bonk (2017) pointed out that searching for high-quality OER among the thousands that are published is a difficult task. Therefore, teachers should consider the quality of the OER they would use by referring to well-known national and international OER repositories, such as the Massachusetts Institute of Technology (MIT), Commonwealth of Learning–OAsis OER and Open Knowledge Repository (all these OER repositories are accessible anywhere). Additionally, assessing and selecting high-quality OER is one of the most challenging tasks while using OER. Therefore, OER can be selected based on several criteria, including licensing, accuracy/quality of the content, interactivity, ease of adaptability, and cultural relevance and sensitivity.
- Professor ID-013 from the online seminar also mentioned that teachers might lack the technical skills to develop their OER. Therefore, to properly create and publish OER, teachers can refer to several national and international authoring tools, such as 101 ppt software and ALESCO Hub, Connexions repository authoring tool or Open Author (all these authoring tools are accessible anywhere), where the learning resources could be simply created via simple clicks and where no specific technical skills are needed.
- During the teaching process, teachers should apply open teaching to engage learners and encourage them to participate in the co-creation of knowledge (Nascimbeni and Burgos 2016). For instance, teachers can ask students to update a given blog related to a specific learning topic using the Baidu encyclopaedia (Wiki pages, for international readers). Additionally, teachers can apply the connectivist learning approach (Goldie 2016) by asking students to write reports as OER on a given topic as well as create new exercises for a specific chapter in an open textbook based on several references and resources. This can help learners gain digital literacy skills (searching, assessing, and identifying online resources) which are fundamental for twenty-first-century literacy. Particularly, teachers can ask students to work on public Tencent documents (Google Docs, for international readers), where they can see one another’s work and progress. This can emphasise peer assessment and reflective practices.
- To facilitate OEP adoption, teachers should select friendly learning tools and technologies that learners are already familiar with. They should also avoid overloading learners by asking them to use too many tools, resulting in inconvenient learning practices for them. Additionally, teachers can refer to open software because by nature it can be modified and adapted to different needs, fulfilling more accessibility requirements than proprietary software (Zhang et al. 2020). For instance, the open source learning management system Moodle was adapted to cover new functionalities, such as detecting at-risk students (Denden et al. 2019).
- Learning is facilitated not only by teachers but also by peers (Hegarty 2015). Therefore, to make the teaching process more interactive, teachers can build open learning communities where the students can openly exchange ideas, create discussions, and collaborate on different tasks. To ensure interactive and open learning communities, teachers should use social networks during the learning process, such as Wechat, QQ, and Sina Weibo (Facebook or Twitter, for international readers). By using these social networks, teachers can share questions related to specific course materials, and students can discuss them to determine specific answers. Consequently, students learn by exchanging ideas and opinions. Furthermore, the jigsaw classroom pedagogy (invented and named in 1971 by Elliot Aronson) can be applied online by dividing the assignment into several tasks and making each team work on a specific task. The teams will use social networks to work together, communicate with one another, and deliver their assignments. This will foster both individual accountability and the achievement of team goals. Additionally, the open learning within social networks can be gamified using Emojis to make the learning process more engaging and interactive. For instance, Saif et al. (2019) used, during the learning process on Facebook, the number of given “likes” on a particular learner’s answer as the score he/she gets for that answer.
- During the learning process using OER and OEP, teachers should act as facilitators of the learning process. For instance, teachers can help their students with their reports by suggesting useful references that they should read. Also, teachers should have an active role in building a trustworthy learning environment by continuously encouraging their students to share their opinions and answers. Hegarty (2015) mentioned that building trust and self-confidence is an important factor in open learning environments so as to achieve excellent learning outcomes.
- Wiley (2013) mentioned that ‘disposable assignments’, meaning assignments which are forgotten right after the course and do not benefit anyone, should be replaced with activities that both teachers and learners can work on and that can benefit others. Therefore, open learning materials delivered by learners (e.g. reports, presentations, videos), under the supervision of teachers, can be collected as open textbooks and uploaded online so that other students and teachers (future generations) can benefit from them. Additionally, learning achievements can be measured within OEP by referring, for instance, to the interaction frequency in open learning discussions as well as the number of finished (uploaded or shared) assignments.
- Professor ID-014 from the online seminar mentioned that traditional paper–based assessments are no longer effective. Therefore, to assess learners in open learning environments, teachers should use project-based assessments based on the OER delivered by learners. In this context, teachers can invite their students to open presentations (where parents and other teachers can attend) of their delivered projects for assessment and grading. This can be achieved via several platforms that support live-video communication, such as Dingtalk and Zoom (Skype and Zoom, for international readers).

### Guidelines for learners

To ensure an active and engaging learning experience using OER and OEP for better learning outcomes, learners should refer to the following guidelines:

- Just like teachers, learners should pay attention to the attributed open licence of each OER to ensure its legal use in their context, as some combination of licenses, for instance, do not allow OER remixing.
- Thousands of OER are published online without knowing the reliability of the authors. Therefore, learners should carefully search for, select, and summarise information while preparing their content (e.g. assignments, presentations, videos, reports) to ensure high-quality OER.
- Learners should remember to attribute open licences to their prepared open learning materials so they can be reused by others as OER.
- To develop their independence and capacity to self-regulate within open learning experiences, learners must develop such skills as behavioural self-regulation and emotional self-regulation. For instance, learners should maintain a positive attitude when facing learning challenges and consider these challenges as new learning opportunities.
- Learners should be collaborative and active in building an open learning community by encouraging their peers and participating in discussions.

Furthermore, professors from the online seminar mentioned that the technical reliability of the internet in some Chinese areas and the complete absence of internet architecture in other areas are major challenges in the midst of this COVID-19 outbreak. Specifically, according to the China Internet Development Report of 2019, the number of Chinese rural internet users reached 225 million, accounting for 26.3% of the total number of internet users in China. Additionally, several professors mentioned that open courses related to prevention against the COVID-19 should be created to increase people’s safety in China and worldwide.

## Urgent applied initiatives to support open and distance education

To further cover the challenges mentioned by professors and education specialists during the two online seminars presented above, several initiatives were created by the MoE as well as Chinese universities and companies to support open and distance education, as follows:

- Several open courses and thematic teaching resources focusing on the epidemic have been produced, including patriotic education, epidemic prevention knowledge, psychological knowledge, and other resources of different disciplines. For example, two schools in Wuhan, the epicentre of the COVID-19 outbreak, have created a variety of educational resources around the epidemic. Additionally, the SLIBNU, in collaboration with the Arab League Educational, Cultural, and Scientific Organization (ALECSO) and the Universidad Internacional de la Rioja (UNIR), has created a series of open resources about COVID-19 protection (see http://sli.bnu.edu.cn/en/Courses/Webinars/Coronavirus_Prevention) in eleven languages: Chinese, English, Arabic, Spanish, Persian, Korean, German, French, Japanese, Urdu, and Bengali.
- The Department of National Textbooks under the MoE has released open versions of teaching books and textbooks for the spring semester of 2020 as well as relevant teaching resources provided by 67 textbook publishers across China, which can be downloaded and used for free by teachers and students all over the country. Additionally, under the unified arrangement of the MoE, the digital teaching resources of the People’s Education Press and its affiliate, Renjiao Digital Publishing Co. Ltd., will be open on the Renjiao Diandu app to primary and secondary school students nationwide. The state has compiled textbooks of three subjects and digital textbooks of the People’s Education Press as well as thousands of video and audio micro-courses synchronised with the textbooks, all contained in the application. The number of users increased by more than 2.3 million, and the number of page views reached 250 million after the app became free for 72 h. According to the statistics of the People’s Education Press, 30 million downloads of elementary- and middle-school textbooks have been observed every day. Similarly, several universities have released several massive open online courses (MOOCs) for learners.
- To increase internet reliability, several Chinese companies – including China Mobile, China Unicom, and China Telecom as well as Alibaba, Baidu, and Huawei – focused on enhancing the provided connectivity services and increasing the internet bandwidth to ensure that 50 million learners can access the cloud learning platform simultaneously and acquire new information without any interruptions.
- To ensure accessible learning experiences, four channels of China Education Television started the open broadcasting of primary- and middle-school classes across the nation, covering 75 lessons on air to provide learning experiences for those in remote areas without internet or without cable TV.

## Discussion, recommendations, and conclusions

The Chinese government considered education as one of its priorities during this COVID-19 outbreak both as a sector that should not experience any discontinuity because of the emergency and as a way to fight the virus itself. Therefore, several initiatives were applied to provide everyone with flexible open and online education. However, despite these strategies and despite the provided guidelines for the better use of OER and OEP, it is seen from this critical situation (COVID-19 outbreak) that several areas related to OER and OEP should be further considered and improved in the future to facilitate OER and OEP application in general and particularly in crisis, resulting in better learning experiences and outcomes. As emphasized by Mrs. Stefania Giannini, UNESCO’s Assistant Director-General for Education: “We need to come together not only to address the immediate educational consequences of this unprecedented crisis, but to build up the longer-term resilience of education systems”. These areas are discussed hereby, and several recommendations for future consideration are presented based on the noted challenges and problems reported by both teachers and learners. Specifically, these recommendations are structured under the five main OER objectives that have been proposed by UNESCO in its recent OER recommendation (2019).

i. *Build the capacity of stakeholders to work with OER*

Both learners and teachers lack the needed skills to create and publish OER. Therefore, several training sessions should be organised to help them to work with OER as well as to deal with the problem of low OER quality (e.g. how to select relevant OER). These sessions should also cover the basic technical skills to produce OER, such as video editing or sound mixing, as well as open licences. They should also be organised as blended learning, where they can be provided first to introduce participants to theoretical ideas and concepts, followed by hands-on workshops, where the participants (teachers and learners) can be practically involved in learning these skills (e.g. teachers working on specific software to edit a video). Furthermore, these training sessions should cover standardised meta-data tagging of a given OER to facilitate its indexing by search engines, hence increasing its visibility to learners.

ii. *Develop supportive open education institutional policies*

Several Chinese universities do not have internal policies on how to deal with OER and OEP. On the contrary, research shows that institutional policies are one of the key aspects to encourage teachers to create and openly publish their resources as OER (Atenas et al. 2019). This can be achieved by, for instance, providing financial incentives for those who contribute to enriching the OER repository of the university. Also, publishing learning materials as OER can be considered by universities as one of the criteria for academic promotion. In addition, decision makers within universities/schools do not know the exact definition of OER and how these can be applied to enhance learning outcomes. Therefore, national seminars in several provinces should be organised to raise awareness about OER and OEP among decision makers and managers of educational institutions. This can result in a rapid increase of knowledge sharing and OER/OEP adoption. Furthermore, while most schools and universities are equipped with reliable infrastructure, several others, especially in remote areas, are not equipped with such infrastructure. This reduces their chances of providing open and online education to their learners. In these cases, governmental policies should be initiated to provide these schools and universities with the needed infrastructure for better teaching and learning experiences to both teachers and learners, respectively.

iii. *Encourage inclusivity end equity through open education*

While the use of distance education is common in China to ensure access to learning for those in rural and remote areas, less attention is normally paid to learners with disabilities. For instance, no OER repository is accessible to learners with disabilities. Also, normally, OER are not specifically published for learners with disabilities. In this context, Zhang et al. (2020) stated in a recent literature review on OER and disability that researchers and educators should pay more attention to developing OER for learners with disabilities, for example by considering different accessibility guidelines, such as web content accessibility guidelines (WCAG 2.0). Furthermore, low-cost technologies which facilitate offline accessibility to OER should be developed, allowing OER accessibility even in regions with low or unstable connectivity.

iv. *Nurture the creation of sustainability models for open education*

The majority of OER projects were funded by the government (e.g. CQC and NCIRSP, as discussed above) only for specific periods (3 to 5 years) without defined long-term sustainability strategies. Therefore, sustainable OER models and strategies should be developed to maintain OER projects and support lifelong learning. Downes (2007) proposed eight possible typologies of OER sustainability strategies, such as sponsorship, where the cost of open content creation and dissemination is covered by sponsors in return for advertising space and promotion. Mengual-Andrés and Payà Rico (2018), on the other hand, stated that sustainability strategies should be carefully studied before their implementation as they depend on several criteria, including cultural and financial situations. To allow each institution to build its own OER and OEP sustainability strategy, the successful experiences of sustainable open education initiatives from around the globe should be broadly shared and discussed among educational leaders and managers.

v. *Facilitate international cooperation*

Bruhn (2017) mentioned that international cooperation within universities is no longer limited to students’ mobility and international agreements. On the contrary, it is now related to deeper strategies to enhance the learners’ experience by providing, for instance, international curricula (Caniglia et al., 2018; Duart, 2011). In this context, Nascimbeni et al. (2020) found that OER can meaningfully support international cooperation on open courses, MOOC development, and virtual mobility practices. In China, while the SLIBNU initiated an international initiative – namely, the Belt and Road (B&R) International Community for Open Educational Resources – to facilitate OER exchange and collaboration in the Belt and Road countries, more specific focus should be put into practice related to OER-centred international collaboration. For instance, the development of international open curricula among universities as well as the translation of high-quality OER from other languages into Chinese (and vice versa). This can reduce the cost and time of developing learning materials, especially in case of emergencies, such as the COVID-19 case.

To conclude, because of the unexpected COVID-19 outbreak, the Chinese educational system is being reformed to maintain the learning process without having to be physically present in classes. This study first presents the education challenges during this emergency and then discusses the application of OER and OEP to overcome these challenges. Particularly, this study presents a generic OEP framework built on existing open practice definitions as well as guidelines for both teachers and learners related to the implementation of OEP for an engaging and active learning and teaching experience and for better learning outcomes. These guidelines are identified based on the challenges highlighted by several experts during two national seminars, organised to discuss online and open education during this COVID-19 outbreak, as well as on several challenges highlighted in the literature by international experts. Finally, this paper discusses urgent strategies that could be applied to support open and online education reform and puts forward some recommendations to foster the adoption of OER/OEP. Future research will focus on presenting a practical experience of using OEP for teaching during the COVID-19 outbreak as well as its impact on learning outcomes.

## Data Availability

Not applicable.

## Abbreviations

OER: Open educational resources
OEP: Open educational practices
MOOCs: Massive open online courses
MoE: Ministry of Education
